# Lightweight Deep Learning Model for Assessment of Substitution Voicing and Speech after Laryngeal Carcinoma Surgery

**DOI:** 10.3390/cancers14102366

**Published:** 2022-05-11

**Authors:** Rytis Maskeliūnas, Audrius Kulikajevas, Robertas Damaševičius, Kipras Pribuišis, Nora Ulozaitė-Stanienė, Virgilijus Uloza

**Affiliations:** 1 Faculty of Informatics, Kaunas University of Technology, 51368 Kaunas, Lithuania; rytis.maskeliunas@ktu.lt (R.M.); audrius.kulikajevas@ktu.edu (A.K.); 2 Department of Otorhinolaryngology, Lithuanian University of Health Sciences, 50061 Kaunas, Lithuania; Kipras.pribuisis@lsmuni.lt (K.P.); Nora.ulozaite@lsmuni.lt (N.U.-S.); Virgilijus.ulozas@lsmuni.lt (V.U.)

**Keywords:** laryngeal carcinoma, substitution voicing, voice analysis, convolutional neural networks, deep learning

## Abstract

**Simple Summary:**

A total laryngectomy involves the full and permanent separation of the upper and lower airways, resulting in the loss of voice and inability to interact vocally. To identify, extract, and evaluate replacement voicing following laryngeal oncosurgery, we propose employing convolutional neural networks for categorization of speech representations (spectrograms). With an overall accuracy of 89.47 percent, our technique has the greatest true-positive rate of any of the tested state-of-the-art methodologies.

**Abstract:**

Laryngeal carcinoma is the most common malignant tumor of the upper respiratory tract. Total laryngectomy provides complete and permanent detachment of the upper and lower airways that causes the loss of voice, leading to a patient’s inability to verbally communicate in the postoperative period. This paper aims to exploit modern areas of deep learning research to objectively classify, extract and measure the substitution voicing after laryngeal oncosurgery from the audio signal. We propose using well-known convolutional neural networks (CNNs) applied for image classification for the analysis of voice audio signal. Our approach takes an input of Mel-frequency spectrogram (MFCC) as an input of deep neural network architecture. A database of digital speech recordings of 367 male subjects (279 normal speech samples and 88 pathological speech samples) was used. Our approach has shown the best true-positive rate of any of the compared state-of-the-art approaches, achieving an overall accuracy of 89.47%.

## 1. Introduction

Laryngeal carcinoma remains the most common malignant tumor of the upper respiratory tract worldwide as reported by Steuer et al. [1]. Literature reports an incidence of around 5 cases per 100,000 inhabitants but National Cancer Institute’s Cancer registry reported 18.3 cases per 100,000 Lithuanian citizens [2]. The most current American Cancer Society estimates for laryngeal cancer in the United States for 2022 are: estimated 12,470 new cases of laryngeal cancer, and predicted 3820 deaths from laryngeal cancer [3]. Although the overall incidence is declining, laryngeal cancer is one of the few oncological diseases in which the 5-year survival rate has decreased over the past 40 years, from 66% to 63%. This may be attributed to more conservative treatment protocols, as well as factors that might delay the patient’s follow-up, mainly—the lack of medical care availability near the patient’s place of residence as described by the report in Journal of Clinical Oncology [4]. Programs that require less specialized medical care and provide patients with reliable follow-up means might help to improve the 5-year survival rate, as well as, increase patient safety during the pandemics [5]. Software that reduces the need for specialized medical care might free up medical facilities for COVID-19 patients. Additionally, this software might reduce the workload of specialized medical personnel and make them available for COVID-19 related tasks. Fewer nonessential trips to outpatient facilities lead to a lower risk of infection during pandemics [6]. This can potentially be achieved without incurring additional costs to the healthcare system.

Chemoradiotherapy and surgery are usually feasible treatment choices for patients with early (stage I-II) laryngeal cancer. The extent of surgery is primarily determined by the tumor’s spread. Depending on the tumor stage, surgical treatment results in locoregional cancer control comparable to that provided by laryngeal radiation or chemoradiation therapy or even higher survival rates, cancer can be achieved for patients who undergo surgical treatment for advanced-stage laryngeal [1].

After laryngeal oncosurgery that may include extended cordectomy (removal of the vocal fold), partial or total laryngectomy patients lose one or even both vocal folds. As a consequence, the voice is generated by a single vocal fold oscillating with the remaining laryngeal and pharyngeal structures or alaryngeal (oesophageal or tracheoesophageal) speech is used. These conditions can be considered as substitution voicing (SV), which is defined as the voicing without two true vocal folds [7]. In SV, involuntary aphonic (unvoiced) segments of speech coexist with rough-voiced ones. Various degrees of speech impairment or even a complete inability to speak after laryngeal oncosurgery are the most important complaints expressed by patients and may lead to their social isolation [8].

During the current pandemic, a lot of specialized medical care facilities and personnel have been dedicated to fighting COVID-19 [9]. This in turn led to delayed diagnostics for primary laryngeal cancer patients and follow-up for patients after treatment [10]. This resulted in the need of more radical cancer treatments and increased patient mortality which otherwise could have been avoided. More than half of laryngeal cancer patients present with stage III or higher at the first appointment. For patients with those stages, total laryngectomy is usually advised for favorable locoregional cancer control and an optimal 5-year survival rate [11]. Total laryngectomy is also performed when the patient is not eligible for conservative techniques like chemotherapy and radiotherapy or in case of their failure. Total laryngectomy provides complete and permanent detachment of the upper and lower airways. This separation causes the loss of voice, smell, xerostomia, and altered taste. Total laryngectomy leads to a patient’s inability to verbally communicate in the postoperative period. Patients after laryngectomy often have to rely on pen and paper or other forms of written text to communicate anywhere from 2 weeks to 6 months after the initial surgery. This is especially troubling during the COVID -19 pandemic when patients have to rely on text messaging to contact their families and have trouble receiving basic social or telemedicine care simply because they can not use the phone by themselves [12].

According to Pereira da Silva et al., loss of voice has a significant influence on the quality of life of laryngeal cancer patients [13]. It has an impact on their communication, social life, and even their ability to keep a job. Furthermore, failure to communicate effectively generates worry, and 40–57% of these people develop a serious depressive condition [14]. As a result, it is critical to give trustworthy voice and speech rehabilitation choices to laryngectomized patients. Because of its ease of use, high success rate in generating speech, and quick training period, vocal prosthesis has become a popular way of rehabilitation [15]. Although effective, all established speech restoration techniques provide patients with distinctly distorted speech patterns, which are perceived as unhealthy by both the patient and society. This is due to the fact that substitution voicing generated speech features high irregularity, frequency shifts, and aperiodicity, together with frequent speech phonatory breaks [16]. This problem often becomes more apparent when the patient has to speak in a loud environment or over the phone [17]. Practitioners often rely on expert opinion on the perceived voice quality measurements, classification, and diagnosis of voice pathology. The problem is that often the procedure is time consuming and can be subject to parameter sensitivity [18]. Latest digitization trends have pushed towards a major improvement in computer-assisted medical techniques. Thus, following established practice, the acoustic prosodic properties of the speech signal have to be modulated by a variety of health-related effects [19], leading to changes in a human voice and the automated detection of pathologies using machine learning has attracted significant medical attention [20].

Many approaches for detecting voice pathology have been proposed in recent research in the above-mentioned literature [21]. However, these systems only attempted to distinguish normal voices from diseased sounds, indicating that there is a research gap in terms of voice illness detection in relation to laryngeal cancer. There are circumstances in machine learning algorithms when speech signals cannot ensure high accuracy and cause time consumption in pathology monitoring systems. As a result, there is an urgent need for a research that highlights the most essential concerns and challenges confronting vocal pathology systems, as well as the importance of illness identification in voice pathology. To our knowledge, not much data on the application of artificial intelligence (AI) technologies for SV assessment exists in the literature (see Section 2). As a result, implementing AI-based models for objective assessment and classification of SV could potentially open up new avenues in research and clinical practice, paving the way for the development of a useful and reliable tool for evaluating SV following laryngeal oncosurgery. Existing deep learning voice analysis approaches generally tend to apply some form of recurrent gates for temporal voice signal analysis, these methods tend to suffer from poor performance and are notoriously difficult to train. It is noticeable, that there is no working AI prototype for SV assessment. As a result, using an AI-based models to objectively assess and classify SV could possibly open up new avenues for study and clinical use. To begin with, a well-designed algorithm might standardize SV evaluation across numerous oncology canters, allowing data sets in different patient groups to be simply compared. The same data sets could be used to improve the algorithm in the future. Instead of the existing methods, but not very efficient already applied methods, requiring prior medical knowledge for signal analysis, we aim to exploit modern areas of machine learning (deep learning) research to extract, measure and objectively classify substitution voicing and speech after laryngeal oncosurgery from the audio signal. The objective estimates obtained can be simplified and used by general practitioners and patients. This would be especially valuable when movement is limited or specialized medical centers are difficult to find, as it was during the peak of the COVID-19 pandemic. Last but not least, AI saves time and does not retire—the knowledge gained via its use is always available and does not expire.

In this paper, we propose using convolutional neural networks (CNNs), generally applied for image classification for the analysis of audio signals by transforming the audio signals waveform into Mels spectrogram and using it as an input in a re-purposed lightweight image classification network. This approach allowed us to achieve the overall accuracy of 89.47% with a simpler network architecture, allowing the approach to be used on computing devices having only Central Processing Unit (CPU) but without a dedicated Graphical Processing Unit (GPU) for the classification of subjects voice pathology.

The paper is structured as follows: Section 2 discusses the state-of-the-art works. The dataset used in this study and the deep neural architecture are described in Section 3. The experimental results are presented and analyzed in Section 4. Finally, the results of this study are discussed in Section 5. The paper concludes with Section 6.

## 2. State of the Art Analysis

A chaotic nature of the substitution voicing signal makes evaluation of substitution voicing improper or even impossible with standard methods of acoustic voice analysis used in clinical settings. Multiparametric models for evaluating voice quality and dysphonia severity are sufficiently reliable and valid because of their correlations to auditory-perceptual evaluation and high reliability and validity in voice pathology detection [22]. Currently, two multiparametric acoustic indices based on sustained vowels and on continuous speech analysis have gained popularity in research and clinical settings to objectively estimate dysphonia: i.e., the Cepstral Spectral Index of Dysphonia (CSID) and the Acoustic Voice Quality Index (AVQI) [23,24]. Both indices may provide reasonable estimates of dysphonia severity and represent valid acoustic metrics for objectifying abnormal overall voice quality [25,26]. However, the use of these indices for assessing SV could be unreliable or technically impossible due to irregular and rather chaotic origin of SV signal. There is no data in the literature about the use of CSID for SV assessment. Only the recent study by van Sluis et al. [27] employed the AVQI to evaluate acoustic voice quality in patients who had undergone total laryngectomy. However, the authors noted that a specific AVQI cut-off value and the discriminative power of this index for SV (tracheoesophageal speech) after laryngeal oncosurgery have to be determined in future research studies. The AMPEX algorithm developed by Van Immerseel and Martens allows automatic reliable analysis of running speech, recognizing regularity patterns for pitch values <100 Hz and differentiating between noise and voicing at low frequencies [28]. Despite the feasibility of AMPEX as a tool for evaluating highly irregular speech has been supported by several studies, this algorithm has not yet gained wider clinical recognition [7,29].

Consequently, to perform automatic voice pathology classification and diagnosis, it is important to obtain reliable signal properties, which is essential for the reliability of the result. The clinical interpretation of vocal features is often conducted before the process of pathology detection [30]. Judging from the analysis of other studies, it is clear that from a technological point of view, many researchers distinguish signal processing functions such as Mel Frequency Coefficients, waveform packet transformations, others use multiple voice analysis tools for a variety of physiological and etiological reasons [31,32,33]. Multiple parameters are used to determine speech roughness, including height, vibration, and flicker, and other methods are often used, such as Harmonic to Noise Ratio, Normalized Noise Energy, and Smooth-to-Noise Ratio [34].

There are two types of possible features to analyze disease impact on voice/speech signal: temporal and spectral [35]. The temporal features (time-domain features) are used to extract and have an easy physical interpretation of a signal (energy, zero-crossing rate, maximum amplitude, minimum energy, time of the ending transient or Log-Attack-Time Descriptor) and are sensitive to articulation. The spectral features (frequency-based features) are obtained by converting the time-based signal into the frequency domain using the Fourier Transform. They might be more efficient for automatic classification because they are not dependent on articulation [36]. The most popular frequency descriptors are fundamental frequency, frequency components, spectral centroid, spectral flux, spectral density, irregularity of spectrum, brightness, etc. [37]. These features can be used to identify changing features in human speech, where the Mel Frequency Cepstral Coefficients are often used in human voice analysis [38]. Methodology from standard speech analysis could be adapted, i.e., using OpenSMILE features [39,40], Essential descriptors, MPEG7 descriptors, jAudio, YAAFE, Tsanas features [41], Wavelet Time Scattering features [42] and Random Forest supervised learning algorithms to detect the symptoms [43] and also to fuse information in the form of soft decisions, obtained using various audio feature sets from separate modalities [44]. In addition, Cepstral Separation Difference could be applied for quantification of speech impairment [45]. Feature extraction using signal-to-noise ratio, harmonic-to-noise ratio, glottal to noise excitation, vocal fold excitation ratio, and empirical mode decomposition excitation ratio methods with Random Forests and support vector machines for classification algorithms can also be used [46].

Alternative approaches could be adopted through Syllable-level Features, Low-Level Descriptor Features, Formant Features, Phonotactic Features with SVM classifier, features extracted using Principal Component Analysis and Linear Discriminant Analysis), SVM, Adaptive Boosting (AdaBoost), K-Nearest Neighbor (KNN) and Adaptive Resonance Theory-Kohonen Neural Network classifiers and the likes. In addition, dimensional reduction techniques such as linear discriminant analysis, principal component analysis, kernel PCA, feeder discriminant ratio, singular value decomposition, and so on are used to find suitable latent variables for classification [47]. Other researchers have taken into account the characteristics of human voice and hearing systems. Aicha et al. [48] used glottal waveform with feature selection using PCA and classification using SVM. Fontes et al. proposed a low-complexity approach using correntropy spectral density [49]. MPEG-7 features are most commonly used for indexing video and audio media and were investigated for this purpose [50]. Hossain et al. have demonstrated that the low-level functions of MPEG-7 sound are effective in diagnosing pathological voice using support vector machines [51]. Vaziri et al. distinguished between a healthy voice and a pathological voice using nonlinear dynamics performance and voice acoustic disturbances [52].

A wide variety of statistical, machine learning based, and other types of algorithms are now widely used for the detection of pathological voice based on the computed acoustic features of the input signal [53]. Pathology classification methods can be sorted into two categories [54]. The first category is “classical” methods, often based on k-nearest neighbor methods and Hilbert-Huang Transforms [55], random forests [56], support vector machines [57], Gaussian mixture models [58], latent Markov models [59], Dynamic time warping [60], discriminative paraconsistent machines [61] and so on. Often these methods are used in combination with traditional features, as illustrated by Ghulam et al., who singled out MFCC from long-term voice voice samples as characteristics and found a significant increase in accuracy in diagnosing pathological voices using the Gaussian mixture model [62]. Other researchers treated voice signals as normal vibration signals when classifying, e.g., Cordeiro et al. calculated the spectral envelope peaks of the voice signal as a function of the classification of pathological voices [63]. Alternatively, Saeedi et al. proposed a pathological voice recognition method based on wave transformation, which calculated the parameters of a wave filter bank using a genetic algorithm [64].

“Modern” side of pathology detection is often related to traditional dense neural networks [65], the more advanced CNNs [66] and very popular recurrent neural networks [67]. Deep learning, which transforms intelligent signal analysis so that algorithms can under certain conditions, theoretically might reach near-medical (expert) capabilities in a variety of voice pathology classification tasks. Chen et al. used 12 Mel frequency cepstral coefficients of each voice sample as row features for their deep learning implementation [68]. Miliaresi et al. suggest to analyze various properties of the voice signal window as low-level descriptors (LLDs) by extracting and analyzing variable-length fragments from the speech signal using the prisms of the main tone, energy, and spectrum [69] and using this data to train the deep learning models. Furthermore, a number of functional elements, such as moments, extremes, percentiles, and regression parameters, will then be applied to each LLD [70], to form a set of aggregate features for a healthy and unhealthy human voice. These statistical summaries can also be combined to form tensors for the training of AI (deep learning) algorithms, where multipath learning and learning transfer could be applied according to the multifunctional LSTM-RNN paradigm [71]. Kim et al. [72] collected features from voice samples of a vowel sound of /a:/ and computed the Mel-frequency cepstral coefficients (MFCCs) using the software package for speech analysis in phonetics (PRAAT), which were used identify between patients with laryngeal cancer and healthy controls. Depending on the features extracted, some authors suggest to an investigation of [53]. Alternatively, it is possible to try to introduce kernel-based extreme learning machines [73] and data preprocessing [74]. Or involves a combination of the k-means clustering-based feature weighting method and a complex-valued artificial neural network [75].

## 3. Materials and Methods

### 3.1. Clinical Evaluation and Equipment

All participants of the study were evaluated by clinical voice specialists performing video laryngostroboscopy (VLS) at the Department of Otorhinolaryngology of the Lithuanian University of Health Sciences (LUHS), Kaunas, Lithuania. VLS was performed using the XION EndoSTROB DX device (XION GmbH, Berlin, Germany) with a 70° rigid endoscope. VLS is routine in clinical practice and did not cause any additional discomfort or delays for the participants.

Speech recordings of the phonetically balanced Lithuanian sentence ‘Turėjo senelė žilą oželį’ (‘The grandmother had a little grey goat’) were obtained using a T-series silent room for hearing testing (T-room, CA Tegner AB, Bromma, Sweden) via a D60S Dynamic Vocal (AKG Acoustics, Vienna, Austria) microphone placed 10.0 cm from the mouth with an about 90° microphone-to-mouth angle. Speech recordings were made at a rate of 44,100 samples per second and exported as uncompressed 16-bit deep WAV audio files.

### 3.2. Dataset

**Figure 1:**
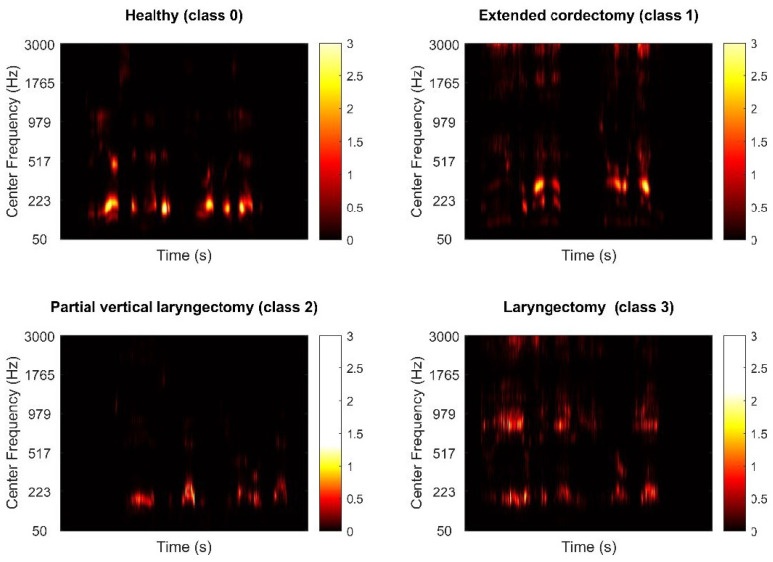
Cochleagrams of each class.

A database of digital speech recordings of 367 male subjects (279 normal speech samples and 88 pathological speech samples) was used. Subjects’ age ranged from 18 to 80 years. The control group comprised 279 healthy male volunteers (mean age 38.1 ± 12.7 years) with the voices evaluated as healthy by the clinical voice specialists. The control group (class 0) subjects had no present or preexisting speech, neurological, hearing, or laryngeal disorders and were free of common cold or upper respiratory infection at the time of speech recording. Furthermore, no pathological alterations in the larynx of the subjects of the normal voice subgroup group were found during VLS. The pathological speech subgroup consisted of 88 (64.1 ± 6.9 years) male patients who used substitution voicing (SV) after oncosurgery. This subgroup included 43 patients after extended cordectomy (class 1), 17 patients after partial vertical laryngectomy (class 2), and 28 patients after total laryngectomy who used tracheoesophageal prosthesis (TEP) (class 3). The pathological speech subgroup patients were recruited from consecutive patients who were diagnosed with the before-mentioned conditions. Speech recordings were obtained at least 6 months after the surgery to ensure a reasonable amount of time for the laryngeal tissue to heal and speech rehabilitation programs to end. A comparison cochleagrams of each class are illustrated in Figure 1. We use the cochleagrams of sound signals for time-frequency analysis and feature extraction instead of the more traditional spectrograms. The signal is initially passed via a gammatone filter, which is designed to mimic the auditory filters found in the human cochlea. The filtered signal is then divided into small windows, with the energy in each window summed and normalized to produce the cochleagram image’s intensity values.

### 3.3. Data Analysis

Table 1 summarizes the voice features captured in the dataset.

**Figure 2:**
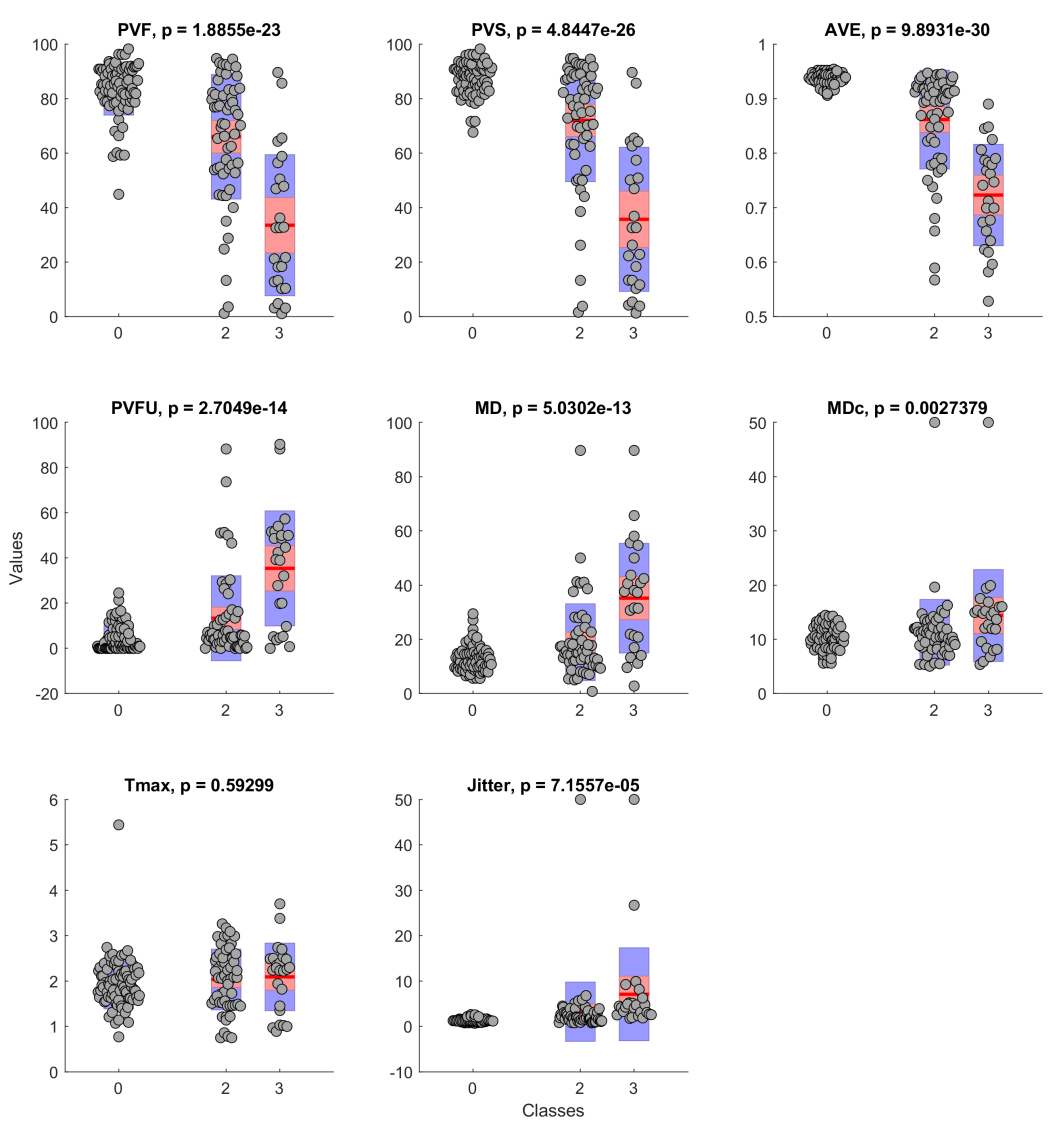
Histogram of feature value distribution among classes with *p*-value from ANOVA test.

Figure 2 shows the histograms of database feature value distributions among classes. The analysis was supported by one-way ANOVA statistical test, which revealed statistically significant differences between classes in PVF (p<0.001), PVS (p<0.001), AVE (p<0.001), PVFU (p<0.001), MD (p<0.001), MDc (p<0.01), and Jitter (p<0.001) values. There was no statistically significant difference in Tmax values.

**Figure 3:**
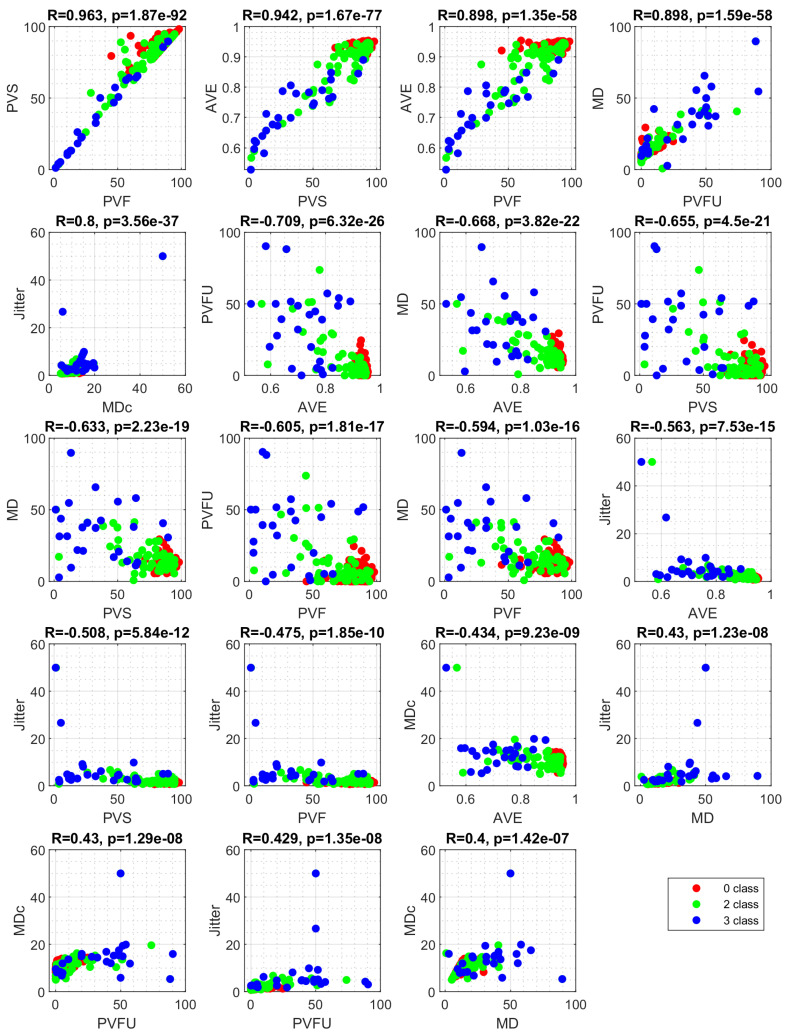
Correlation between feature values among classes. Correlation value (*R*) and its significance (*p*) are given. The plots are arranged by decreasing statistical significance of the determination coefficient ($R^{2}$). Only plots with significant correlations are shown.

Figure 3 shows the correlation between feature values among classes in database. The strong correlation was found between PVS and PVF (R=0.963, p<0.001), PVS and AVE (R=0.942, p<0.001), and MD and PVFU (R=0.898, p<0.001). This shows a strong co-linearity property in the database, which makes it difficult to use for training classical machine learning models [76].

### 3.4. Architecture

**Figure 4:**
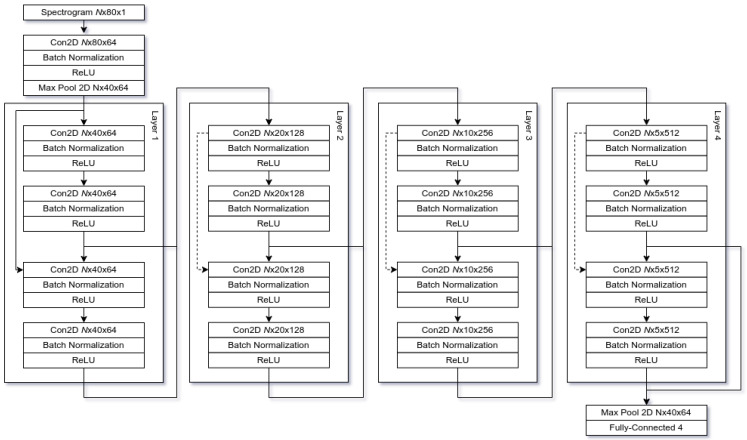
Our approach, here *N* is the sequence length, dashed lines are skip connections.

Figure 4 shows our approach deep neural network architecture. Our approach takes an input of Mel-frequency spectrogram (MFCC) as an input with a total of 80 coefficients. Therefore, given a waveform, the converted MFCC spectroctrogram gives an input of N×80×1 where *N* is the sequence length. Each of the layer blocks starts with a convolutional network with stride 2, this reduces the input dimensionality by half. Layers 2, 3 and 4 internally contain skip connections (dashed lines), these allow for a better gradient flow. The fourth and final layer is then connected to fully-connected that has 4 neuron output, each of the neurons is belongs to one of four voice classes. The network is trained using initial learning rate of $lr=10^{-4}$ with the batch size of n=16, to reduce memory requirements training was performed on half-precision floating points. Because the sequence length between the audio files was not equal the each of the batch audio files have been padded with zeroes to equalize the sequence length. The network was trained for 3000 epochs using Adam optimizer [77] and cosine annealing with warm restarts every 500 epochs, which would adjust the learning rate in the range of $lr=[10^{-7};10^{-4}]$, cosine annealing was chosen for it has demonstrated the ability to achieve better recall rates due to potentially jumping out of local minimums [78]. The hyper-parameter values were chosen during empirical experiments. Over-fitting was avoided by employing an early stopping process and batch normalization.

### 3.5. Implementation

**Figure 5:**
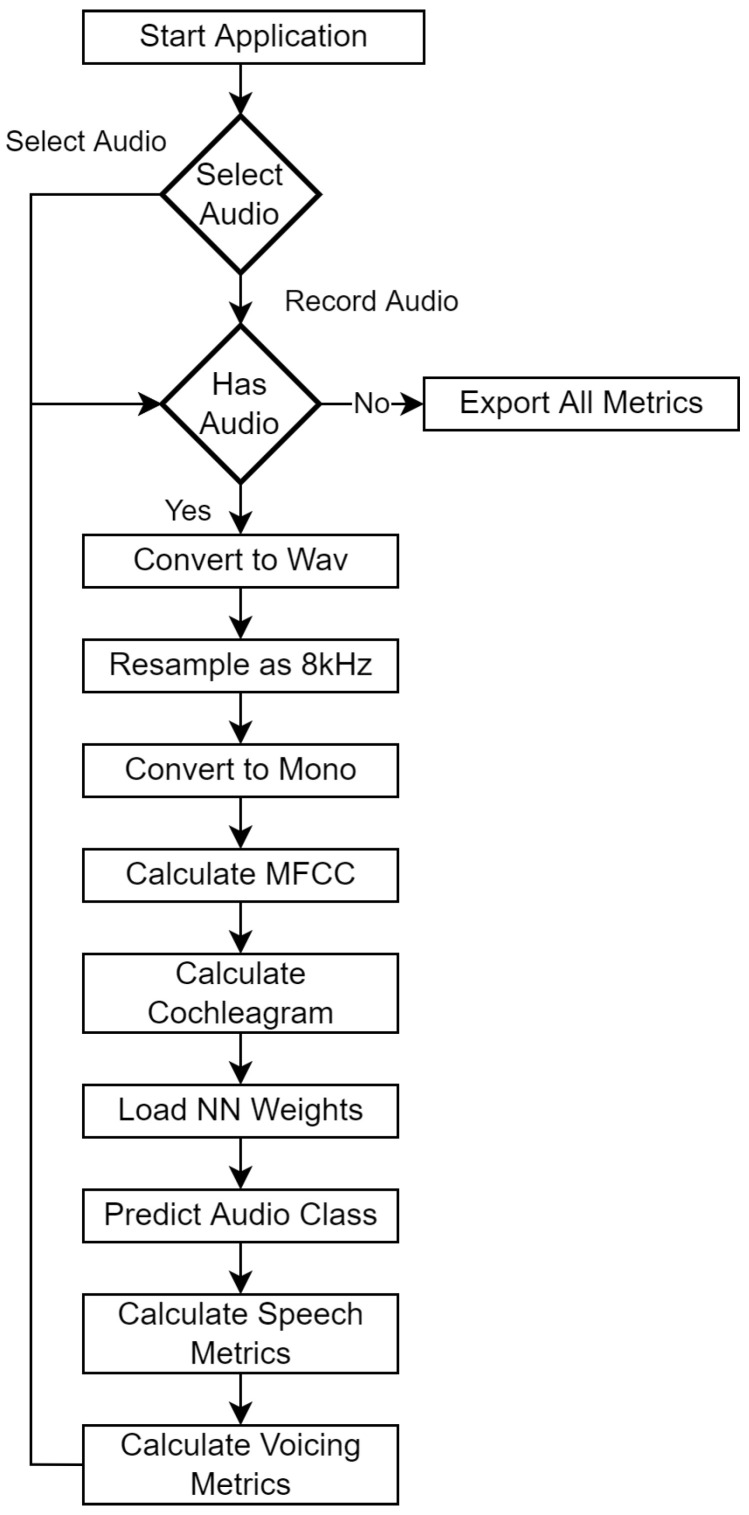
Architecture of the system.

**Figure 6:**
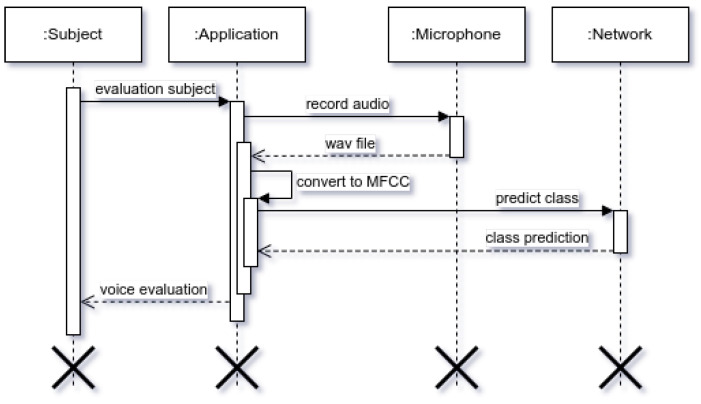
Voice evaluation sequence diagram.

**Figure 7:**
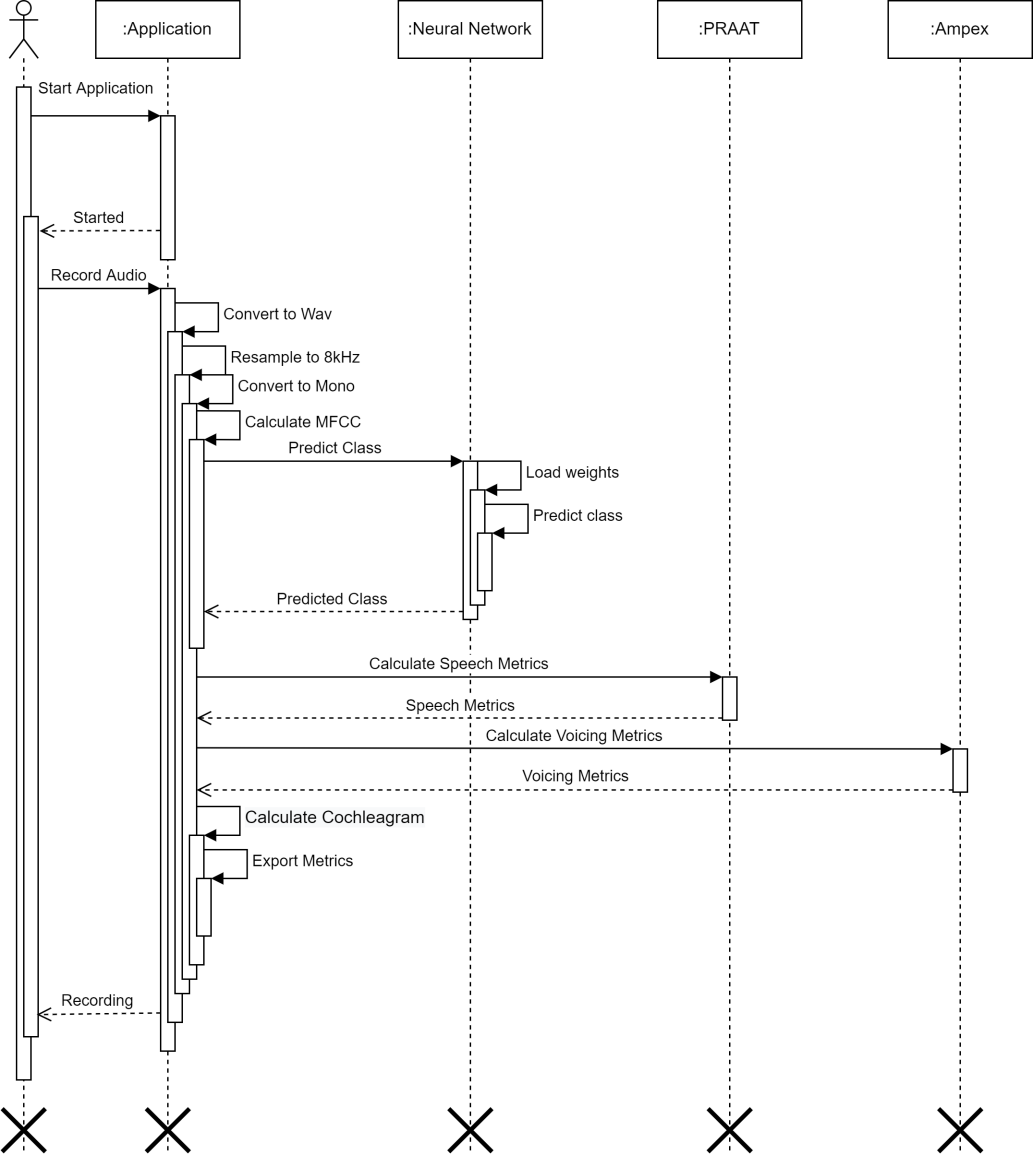
Composition of the voice evaluation sequence processes.

In Figure 5, Figure 6 and Figure 7 we can see how our approach works for evaluating subject’s voice class. In order for the subject to evaluate their voice, firstly they need to make a voice recording using their microphone, the audio waveform is sampled using mono-channel 8000 Hz sampling rate (as 8 kHz still retains voice information (as stipulated by most standards, including telephony), a down-sampling (from 44 kHz to 8 kHz) was performed to optimize the required quantity of data and reduce network overhead while taking VRAM limits into account.). After the voice waveform is recorded, it is then converted into Mels-frequency diagram using 80 coefficients. Normally, this would be around 40 MFCC samples, however the system kept too little information in our situation (as substitution voicing loses a lot of information in relation to “healthy” speech), therefore 80 MFCC samples was the best determined option. The MFCC spectrogram is then used as an input in our neural network, where one of four classes are predicted: healthy, one-voice fold pathology, two-voice fold pathology, and finally nonspecific voice pathology.

## 4. Experimental Evaluation and Results

### 4.1. Setup

To test our minimalistic CPU optimized approach, we have used augmented the dataset and used 147 recordings containing no voice pathology (normal voice), 111 voice recordings of mass lesions of one single vocal fold, 57 recordings of mass lesions in both vocal folds, and finally 67 recordings containing nonspecific voice pathology from the dataset collected in Lithuanian University of Health Sciences (see Section 3.2). The training set is divided using 80:20 rule, where 80% of the recordings of each class separately are used for training, and the remaining are used for validation. Additionally, because the dataset is highly unbalanced, we have dropped the data points in classes that have an excess of data, this allows all classes to have an identical amount of data samples, reducing the probability that the network will overfit using any of the underlying classes. To evaluate and compare our approach versus state of the art, we have used confusion matrices as they best reflect the results in multiclass problems by allowing us to evaluate true-positive versus false-positive rates.

### 4.2. Metrics

We used accuracy, precision, recall and F1-score as fitness measures. These are defined as follows:

(1) $$Accuracy=\frac{\mathrm{TP}+\mathrm{TN}}{\mathrm{TP}+\mathrm{TN}+\mathrm{FP}+\mathrm{FN}}\times 100\%$$

(2) $$Recall=\frac{\mathrm{TP}}{\mathrm{TP}+\mathrm{FN}}$$

(3) $$Precision=\frac{\mathrm{TP}}{\mathrm{TP}+\mathrm{FP}}$$

(4) $$F1=2*\frac{Precision*Recall}{Precision+Recall}.$$

where TP (true positives) is the number of voice pathology samples that were labeled correctly, TN (true negatives) is the number of non-pathotology voice samples that were labeled correctly. FP (false positives) is the number of voice pathology samples that were labeled incorrectly as being not voice pathology samples, and FN (false negatives) is the number of not-pathology samples that were miss classified as pathology samples.

### 4.3. Results

**Figure 8:**
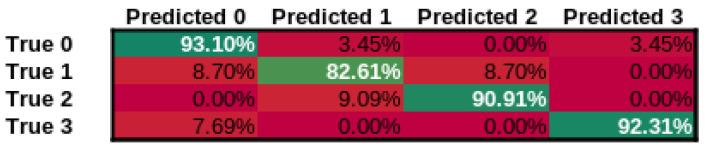
Confusion Matrix for our approach.

**Figure 9:**
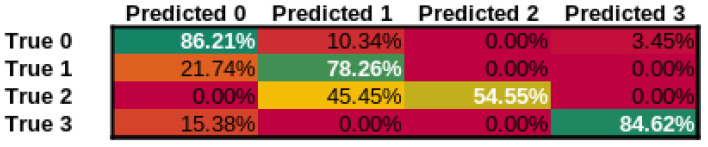
Confusion Matrix for ResNet-101 model.

**Figure 10:**
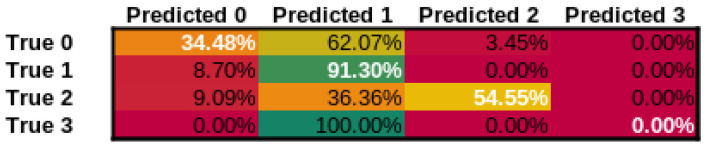
Confusion Matrix for Wav2Letter model.

**Figure 11:**
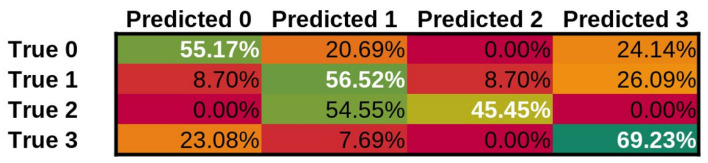
Confusion Matrix for M5 model.

In addition to our approach, we have tested three additional approaches, ResNet-101 [79], a state-of-the-art image classification network, Wav2Letter [80] and M5 [81] as state-of-the-art audio classification networks using the identical training procedure and datasets. The confusion matrices for our approach can be seen in Figure 8, for ResNet-101 can be seen in Figure 9, Wav2Letter in Figure 10, and finally M5 confusion matrix can be seen in Figure 11. Here Class 0 represents normal voice; Class 1 represents SV after cordectomy; Class 2 represents SV after partial laryngectomy; Class 3 represents SV using TEP. As we can see, our approach has shown the best true positive rate of any of the compared state-of-the-art approaches. Giving an overall accuracy of 89.47%.

**Figure 12:**
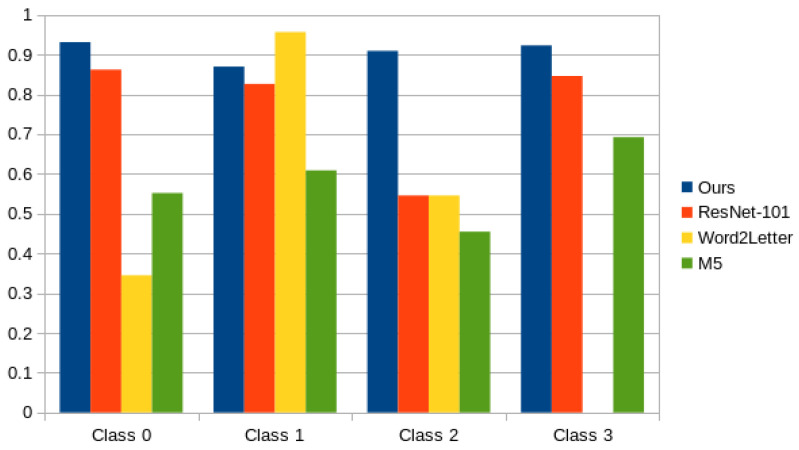
Comparison of performance between different models: ResNet-101, Word2Letter, M5 and our model.

In Figure 12 we can see the model accuracy comparison side-by-side for each of the approaches broken down by class, additionally we can see our approach result breakdown in Table 2, as we can see, the accuracy for all of each of the individual classes is above 90%.

**Figure 13:**
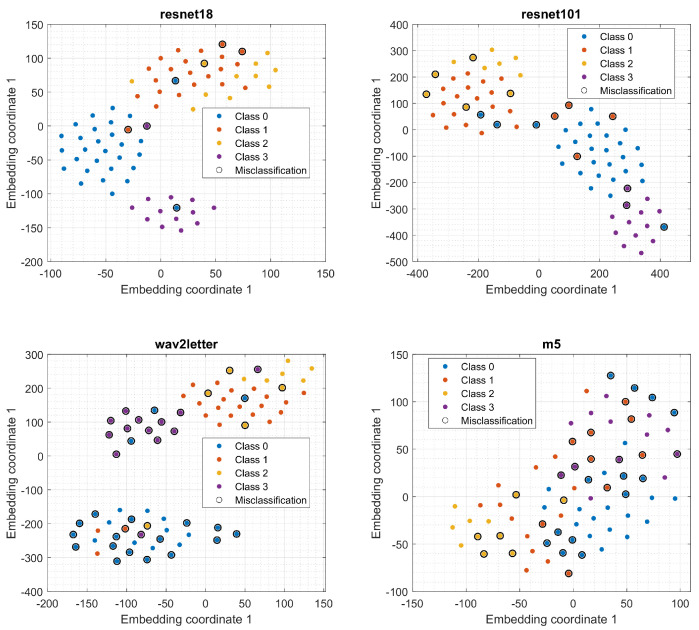
Comparison between t-SNE embeddings of different model predictions.

To analyze the predictions of models more precisely, we used t-distributed stochastic neighbor embedding (t-SNE), a statistical method for visualizing high-dimensional data by mapping it to a two-dimensional embedding. The results are presented in Figure 13. It shows that the classes are well separated while the miss-classifications using the best model (resnet18) are few.

## 5. Discussion

This work provides a technique for automatically assessing if a voice is healthy or whether its quality has changed owing to a pathological condition. Because these spread swiftly, automatic detection is necessary, yet it is frequently underestimated. Machine learning is making a significant contribution to illness diagnosis and early detection in cardiology, pulmonology, liver tumor segmentation, and other fields of healthcare. As a consequence, machine learning might be employed effectively in a computer or mobile healthcare system to automatically identify and detect irregularities in a person’s speech for early diagnosis.

For the study of speech audio signals, we propose employing well-known CNN models that have been used for image classification. Our method uses a Mel-frequency spectrogram (MFCC) as an input to a deep neural network architecture while achieving very good classification results. Our outcomes demonstrate that a deep learning model after training using a pathological speech database, voice alone might be utilized for common vocal fold illness identification using a deep learning technique. This AI-based technique might be therapeutically effective for screening general vocal fold illness using the voice. A brief assessment and a general health examination are part of the strategy. It can be used during telemedicine in places where primary care facilities do not have laryngoscopic capabilities. It might aid physicians in pre-screening patients by allowing invasive exams to be done only in situations involving issues with automatic recognition or listening, as well as expert evaluations of other clinical examination findings that raise concerns about the existence of diseases.

The biggest issue that each patient suffers, especially those who live in distant areas, is the lack of physicians and care in emergency circumstances. As a result, there is a need to provide a new framework in such remote locations by utilizing telecommunication means and artificial intelligence methods for automated voice analysis in the context of remotely-provided telehealth services [82]. Telehealth is a successful paradigm for diagnosing and treating voice issues in remote locations, as an alternative to face-to-face consultations. Telehealth consultations have been found to contribute to medical diagnosis for a variety of vocal problems, with diagnostic decision outcomes comparable to in-person consultations [83]. There are several instances in which patients require long-term monitoring. In this sense, the provision of continuous monitoring is critical. Because laryngeal cancer is a potentially fatal disease, new and effective methods for laryngeal cancer early detection are desperately needed. The method provided in this study enables an effective and noninvasive way for diagnosing laryngeal carcinoma.

## 6. Conclusions

In this paper we used cutting-edge deep learning research to objectively categorize, extract, and assess substitution voicing after laryngeal oncosurgery from audio signals. For the study of speech audio signals, we propose employing well-known CNNs that have been used for image classification. Our method uses a Mel-frequency spectrogram as an input to a deep neural network architecture. A database of 367 male participants’ digital voice recordings (279 normal speech samples and 88 abnormal speech samples) was employed. Our method has the highest true-positive rate of any of the assessed state-of-the-art methods, with an overall accuracy of 89.47%.

## Figures and Tables

**Table 1:** Summary of voice features.

Feature	Description
PVF	Percentage of voiced frames
PVS	Percentage of voiced speech frames
AVE	Mean voicing evidence of voiced frames
PVFU	Percentage of unreliable voiced frames
MD	Average F0 modulation
MDc	MD only in frames with a “reliable” F0 estimate. Vocal frequency estimate F0 is considered reliable if it deviates less than 25% from the average over all voiced frames.
Jitter	F0-jitter in all voiced frame pairs (=2 consecutive frames)

**Table 2:** Our result approach breakdown by class.

Class	n (Truth)	n (Classified)	Accuracy	Precision	Recall	F1 Score
**0—normal voice**	30	29	93.42%	0.93	0.9	0.92
**1—SV after cordectom**	21	23	92.11%	0.83	0.9	0.86
**2—SV after partial laryngectomy**	12	11	96.05%	0.91	0.83	0.87
**3—SV using TEP**	13	13	97.37%	0.92	0.92	0.92

## Data Availability

The dataset used in this study is available from the corresponding author upon reasonable request.
